# Editorial: Saliva used as biological fluid to detect neurodegenerative and neurodevelopmental diseases

**DOI:** 10.3389/fnins.2023.1141376

**Published:** 2023-02-28

**Authors:** Gianluca Tartaglia, Stephen Connelly

**Affiliations:** ^1^Department of Biomedical, Surgical and Dental Science, University of Milan, Milan, Italy; ^2^UOC Complex Operative Unit of Dentistry and Maxillo-Facial Surgery, Foundation IRCCS Ca' Granda Ospedale Maggiore Policlinico, Milan, Italy; ^3^San Francisco Veterans Affairs Health Care System, Department of Oral & Maxillofacial Surgery, University of California, San Francisco, San Francisco, CA, United States

**Keywords:** saliva, neurodevelopmental disease, neurodegenerative disease, diagnostic, autism spectrum disorders

Neurodegenerative and neurodevelopmental diseases represent neurological disorders that variably affect individuals and produce negative consequences, such as altered social interaction, restricted/repetitive behavior, and other medical and mental health conditions that result in lifelong functional and social impairments with high economic and social costs (Livingston et al., 2020). Elderly members of our increasingly aging population are more vulnerable to these neurological disorders and the high individual and societal costs are obvious (Bieleninik and Gold, 2021). Given the present and future challenges, it is important to focus our efforts on the early diagnosis and treatment of neurological diseases, as the initial biochemical pathophysiological drivers of these disorders occur far earlier than symptoms appear, at which point treatment alternatives are sparse and often futile (Bjerke and Engelborghs, 2018; Ausó et al., 2020). Thus, the establishment of temporally relevant and robust diagnostic methods in the form of disease-specific biomarkers is urgently needed to impact disease development and progression. The ideal biochemical biomarker(s) should be low-cost and easily accessible. With those parameters in mind, saliva represents a huge opportunity. The sampling of saliva is non-invasive, time-efficient, and offers the possibility of biomarker identification and surveillance on a continual basis for a variety of systemic diseases, for which specific salivary biomarkers have already been identified and put to use. Saliva is a very complex matrix of fluids and proteins that is an accurate recapitulation of the proteins and other molecules present in circulating plasma. In the past, this complexity has presented challenges for those attempting to accurately measure its components. However, despite those past challenges, salivary bioscience has made significant strides in both academic and industry settings as recent technological advances in the measurement of saliva components have opened up a window of opportunity to use saliva to detect and track disease.

This Research Topic on saliva-based diagnostic approaches for detecting and tracking diseases was launched with the intention of encouraging both the academic and industrial communities to continue developing and refining technologies that will make salivary biomarker detection ever more reliable and relevant. As acceptance of this mode of systemic sampling grows, it is anticipated that robust clinically relevant platforms will become commercially available. With regard to improving commercial viability, it will be important to establish salivary biomarker measurement as a “gold standard,” with the same validity and acceptability as the common blood-based lab tests that are routinely used today. The advantages of salivary bio-sampling are clear: it is low-cost, simple, non-invasive, and offers high levels of sensitivity and specificity for the detection and surveillance of disease. A saliva-based platform would be particularly advantageous for studying diseases that require large sample sizes for statistically valid disease detection.

This Research Topic includes a selection of original articles and systematic reviews about the salivary bioscience related to neurodegenerative and neurodevelopmental diseases, their determinants, and the impact of these conditions on quality of life. Additionally, the Research Topic features articles that discuss salivary biosensing as applied to preventive and therapeutic applications of neurological disease. We have a variety of publications: one mini review, two original feasibility studies, and two original research articles based on the technological aspects of neurological biomarker detection.

The mini review entitled, “*Saliva Based Diagnostic Methodologies for a Fast Track Detection of Autism Spectrum Disorder: A Mini-Review*” discusses the rising prevalence of autism spectrum disorders (ASD) and presents a compelling argument in support of salivary biomarkers to diagnose this complex disorder, which currently relies solely on behavioral assessments (Sharma et al.).

This mini review is followed by two original research articles. The first, entitled “*Salivary MicroRNA Profiling Dysregulation in Autism Spectrum Disorder: A Pilot Study*”, demonstrates how saliva can help clinicians support the ASD diagnostic process through the standardization of conditions used to isolate biomarkers that can robustly detect disease in a more simple way (Kalemaj et al.). The second, entitled “Salivary Inflammatory Biomarkers are Predictive of Mild Cognitive Impairment and Alzheimer's Disease in a Feasibility Study,” provides evidence for the feasibility of saliva as a valuable source of biomarkers for the early detection of cognitive impairment in individuals on the AD continuum and potentially other neurodegenerative diseases.

The two remaining studies, “*Non-Invasive Diagnosis and Monitoring Tool of Children's Mental Health: A Point-of-Care Immunosensor for IL-6 Quantification in Saliva Samples*” (Cruz et al.) and “*Multisite Dopamine Sensing with Femtomolar Resolution Using a CMOS Enabled Aptasensor Chip*” (Sessi et al.) provide examples of how point-of-care (POC) biosensors can help societies fast track patient needs in the early diagnostic process. For instance, preliminary analysis of retrospective PoliBiobank data (UOC Unità operativa Complessa Chirurgia Maxillo-Facciale e Odontostomatologia, Fondazione IRCCS Ca Granda, Ospedale Maggiore Policlinico, Department of Medicine, Surgery and Dentistry, University of Milan) demonstrates that miRNA and inflammatory molecule biomarkers in saliva samples collected from healthy individuals and those diagnosed with neurodegenerative and neurodevelopmental diseases, using traditional techniques, can be detected *via* real-time PCR and the MSD platform, respectively (unpublished data).

Finally, as we highlighted in Goldoni et al. (2022), our hope is that readers will find the diversity of the content published in this Research Topic interesting and valuable. We are happy to present reviews and original research articles for this promising area of research, ranging from population-based studies to patient-centered clinical studies evaluating the effectiveness of treatment and preventive regimens.

## Author contributions

All authors listed have made a substantial, direct, and intellectual contribution to the work and approved it for publication.
